# Hospital Expenditure and Efficiency

**Published:** 1905-04-01

**Authors:** 


### HOSPITAL EXPENDITURE AND EFFICIENCY.

### MR. SYDNEY HOLLAND'S REPLY AND ITS ANSWER.

We have received a letter from Mr. Sydney Holland and have submitted it to the test of facts. Mr. Holland's reply is printed in ordinary type and our comments follow each paragraph and are printed in italics. We are thus able to finally dispose of the discussion in one article.

### Western Infirmary, Glasgow.

Mr. Sydney Holland writes: The figures I gave were those of the hospital of which Dr. Mackintosh is the superintendent, not of any other. I did not refer to the Royal Infirmary, Edinburgh. I repeat them, and shall be glad to own myself wrong if they are inaccurate, as you say. My humble gibe about porridge has raised the clans, and has been taken too seriously. But I repeat that none of us can get English patients to eat porridge—more is the pity.

The Western Infirmary get their beef at 3¾d. per lb.—we have to pay 4⅝d. to 5½d. I said meat—I meant beef and quoted the figures of beef. I was under the impression that the Western Infirmary resembled the Edinburgh Royal Infirmary in giving no medicine to out-patients, but Dr. Mackintosh set me right about this. But even if the figure you give of 40,000 prescriptions made up is correct, it is a very small one as compared with the "London." They get their water free—we pay £500. Their coal is 12s. 6d. as against our 16s. 10d., and 8s. 6d. for furnace coal as against 21s. 8d. Their rates and taxes are very low; ours are £1 3s. 8d. per bed. The report I received gave them as £35 only; and it is before me as I write. On page 15, which you quote, there are mysterious words such as "Teinds," "Crop 1903," "Ground Annual," and "Crown Duties." If these are equivalent to our rates and taxes my total must be increased to the sum you name, £237 against £934 at the "London." They get their telephones free; we pay £325 a year. We have to have two trunk lines and a telephone exchange in the building, and two women are constantly employed. Their medical salaries are £2 5s. per bed; ours are £7 10s. Their stokers work a 12 hours shift; ours will not. They give no pensions at present, so my statement on this point remains accurate, whether this is due to the comparative newness of their hospital or not; our pensions are £2,279.

*Mr. Holland's method of picking out one or two items from a great number at one institution, and then to compare them with those at another where they may cost more or less, according to the view advocated, must prove valueless for practical purposes. In the same way and for the same reason the selection of one hospital because it happens to be lowest in its cost per bed without regard to circumstances and efficiency can form no safe guide as a test of the relative merits of two systems of administration. What good end can be served either by making comparisons if one always selects the lowest price of a particular article without giving the details? Thus, Mr. Sydney Holland says the Western Infirmary get their beef at 3¾d. per lb.,* i.e. *the lowest price paid at that institution, where as much as 4¼d. per lb. is paid for beef. Why leave mutton out altogether, which costs 8d. per lb. in Glasgow, whereas they get it at the "London" for 5d. or 6d. Besides, economy in purchase is only one aspect of the problem; economy in use is probably of greater importance still.*

Vegetables, Puddings, and Massage.

They give no green vegetables or puddings to patients, and our dietary is certainly a more generous one in many ways. Contradicting my statement that no massage is given to out-patients, you write that £47 was spent on massage, but this was for in-patients. They do not massage any out-patients. The attendances in our out-patient massage department numbered 13,000. Their nurses are paid less, and have less off-duty time, and they have not so many per bed.

Of course, all my statements were made on the report of the secretary of the London Hospital who had visited the hospital and made careful inquiry. I have not visited the hospital myself. And my remarks were not made in any spirit of hostile criticism of the Western Infirmary, but merely as a comparison of how much cheaper things can be done and are done at that hospital than in London. I am sure of one thing that no London hospital can be managed as cheaply as a Scotch hospital is, and I am sure also that there is much to be learnt from Scotch hospitals in their microscopic attention to every detail of expenditure.

*Again, Mr. Holland states that the Western Infirmary give no puddings to patients; but Dr. Mackintosh pointed out on page 10 of his paper that the patients there for dinner "have soup and, in addition, either boiled beef or mutton, with potatoes or bread, quite apart from such articles of food as are ordered by the doctor—for instance, chop, chicken, custard, and extra milk." Had Mr. Holland read the paper carefully the inaccuracy of the statement he makes,* i.e., *they give no puddings to patients, must have been clear to him. Again, as a matter of fact, they do give massage to out-patients at the Western Infirmary, where the treatment is called for. As to the nurses per bed, that raises an important point which is likely to engage the attention of hospital managers pretty closely in the near future. It has not yet been decided what is the maximum and minimum number of patients to nurses which should form the standard of the best-administered hospitals in this country.*

*We publish in another column a letter from a gentleman of over thirty years' experience in the dispensaries of London hospitals, which shows that Mr. Holland's views in regard to surgery and dispensary and the preparation of drugs is not borne out by practical experience.*

Guy's Hospital.

And now, as to Guy's. Sir Henry Burdett's argument has been that given a medical superintendent down would go the cost per bed in London hospitals by £20 per year!

*Sir Henry Burdett's words are: "A medical superintendent in charge of every department who would keep the expenditure practically uniform or nearly so, within a couple of years should easily reduce the expenditure of most London hospitals by £20 per occupied bed." If Mr. Holland were to spend a couple of days at the Western Infirmary, Glasgow, with Dr. Mackintosh and thoroughly master the system there, he would probably agree with this view.*

Reductions in Cost.

I carefully avoided at the Hospital Associations' meeting making any comparison between Guy's and any hospital with which I am connected. But I showed conclusively, and I am sure the meeting was with me, that the result of having a medical superintendent at Guy's has *not* been to reduce the cost of anything at that hospital below the cost of several other hospitals, which have no medical superintendent, and certainly not £20 a bed below hospitals without a medical superintendent. I said, and repeat it, that the statistics given in . . . Burdett's Annual . . . are inaccurate as regards Guy's.

*Mr. Holland showed nothing "conclusively," as he claims, as the verbatim report of the meeting shows. His statement was that "Guy's Hospital in Provisions, in Alcohol, in Surgery, and in Domestic Service was behind other London hospitals"; but in* The Hospital *of the 18th ult., page 451, we proved this statement to be incorrect by giving the actual figures under the various headings mentioned.*

Expenditure in Alcohol.

1. *In Alcohol.*—It is the custom in the Guy's accounts to show the gross total of board of residents and of nursing staff without specifying the items. This is overlooked by Burdett's guide, and that guide takes the sum put down for alcohol for patients only!

*This is so, but the same point might be urged in regard to other hospitals where the accounts do not separate the items sufficiently. The Editor has made this point for years in regard to many items which have in consequence been subsequently analysed correctly in the accounts of various hospitals. Mr. Holland cannot, however, have it both ways, for if the item of alcohol is increased at Guy's, item No. 4, Provisions, must be proportionately decreased.*

Domestic Expenses.

2. *Domestic Expenses.*—We now know, since the Report of Sir Edward Fry's Commission, that Guy's gets 10 of its residents lodged, served, and provided with fuel, light, and attendance by the college without charging the hospital. Consequently, comparisons with other large hospitals under the headings "domestic," "rent," and "salaries" are vitiated and inaccurate. But, even taking the figures as they stand, Bart.'s beats Guy's; and if the Guy's were altered and debited with the above expense, Guy's would be still further behind.

*The position is not correctly stated. The residential college occupies a piece of land which cost the hospital £5,500. At 3 per cent. the rental would be £165 per annum. The school pays no rent, but lodges the ten residents, there being no accommodation for them in the hospital. The ground lease expires in 1935, when the hospital will come into property*

*worth £26,500. This is good business for the hospital, and Sir Edward Fry's Commission by its findings disposed of this point.*

Central Power Scheme.

More than this, on looking at back accounts of Guy's I notice that while domestic expenses were £32 a bed in 1901, they went down in 1902 to £25, and in 1903 to £20 16s., a drop of £11 in the three years. But I also notice that establishment went up £9 10s. in the same years—*i.e.* from £19 10s. per bed in 1901 to £29 in 1903. In 1902 Guy's started their excellent "Central Power Scheme," and these figures suggest to me that perhaps expenses which were formerly entered under the heading "domestic" have been transferred to establishment. I have no doubt quite rightly so; but, if this be so, it again proves that Burdett's Annual is unfair in making the comparisons. In establishment Guy's is £16 above the average of the 12 hospitals, but I have no doubt that a saving is made in other ways in fuel or lighting. I am only concerned to point out how fallacious and misleading these comparisons are.

*The following comparative statements for the years in question show that Mr. Holland is quite wrong in his contention here. The figures prove a general decrease in practically every item, and not on any particular item, which would be effected by the transfer due to the Central Power Scheme. A reduction of £13 per bed in Domestic Expenditure in four years strongly supports the view that great reductions are effected by the control of a medical superintendent.*

Guy's Hospital.

| — | 1901. | 1902. | 1903. | 1904. |
|---|---|---|---|---|
| *Domestic Expenses—* | £ | £ | £ | £ |
| *Renewal of Furniture* ... ... | 786 | 635 | 632 | 1,359 |
| *Bedding and Linen* | 1,452 | 504 | 914 | 595 |
| *Hardware, Crockery, etc.* ... ... | 820 | 1,047 | 764 | 870 |
| *Washing* ... ... | 2,843 | 2,221 | 1,802 | 1,350 |
| *Cleaning and Chandlery* ... | 1,781 | 1,821 | 1,252 | 939 |
| *Water* ... ... | 494 | 401 | 162 | 154 |
| *Fuel and Lighting* | 5,541 | 4,544 | 4,191 | 3,878 |
| *Uniforms* ... ... | 1,241 | 900 | 617 | 533 |
| *Sundries* ... ... | 78 | 66 | 58 | 77 |
| *Total*... ... | 15,036 | 12,139 | 10,392 | 9,755 |
| | £ | £ | £ | £ |
| *Cost per Bed* ... | 32 | 25 | 20¾ | 19 |

Administration.

3. *Administration.*—Guy's is represented in Burdett's Annual to have a very low cost of administration. But the editor has failed to notice that no less than £2,141 of administration expenses are put aside under "Sustentation Fund"—all of which expenses come into the accounts of other hospitals.

*This item should have been shown under Appeals and Festivals instead of under Extraordinary Expenditure. In the Report for 1904 Guy's have so amended their accounts.*

Medical Superintendent's Reductions.

These inaccuracies render the comparisons with other hospitals of no value. But, apart from them I maintain that Guy's—an admittedly good hospital—completely disproves Sir Henry Burdett's statement, that a medical superintendent can reduce the cost per bed by £20 over hospitals without such an officer.

*We have shown that the points raised are not inaccuracies, and that in one item, Domestic Expenses, the medical superintendent has secured a reduction in four years of £13 per bed, a fact which supports Sir Henry Burdett's statement. See also reply "Generally" below.*

Provisions.

Take other items—

4. *In Provisions.*—It is admitted that Guy's is £1 12s. 6d. per bed higher than the average of the 12 hospitals with schools. But be it noted that Guy's is £2 18s. per occupied bed higher than St. Thomas's, where there is no medical superintendent.

*In dealing with No 1, Alcohol, we show that these figures would be reduced in favour of Guy's if the expenditure on Alcohol supplied to the residents and nurses were to be deducted.*

Surgery and Dispensary.

5. *Surgery and Dispensary.*—"Bart.'s beats Guy's at this, and I dare say other hospitals do too, but "Bart.'s" has no medical superintendent. This was the item of expense where Dr. Mackintosh claimed that a medical superintendent was so much more valuable than a layman. I think I am right in stating that St. Mary's, with an able medical superintendent, spends more than any of the 12 hospitals on surgery and dispensary. I do not say that it spends too much, but I insist that having a medical superintendent does not reduce even this item of expenditure.

*One swallow does not make a summer. The medical superintendent at Guy's has succeeded in reducing the cost per unit for surgery and dispensary much below the average cost at the 16 largest London hospitals. Before Bart.'s can be properly compared with Guy's we must wait for the erection and opening of the modern and up-to-date out-patient department now in course of construction. St. Mary's Hospital does not in fact possess a medical superintendent in charge of the whole internal administration of the hospital. Mr. Holland is therefore wrong on this point.*

Not Comparable.

6. *In Salaries and Wages.*—The Royal Free and King's College are lower than Guy's, and neither of these hospitals has a medical superintendent.

*Having regard to the number of beds occupied, a comparison like that drawn by Mr. Sydney Holland is of no value. Mr. Holland ignores the fact, however, that although Guy's spends on Salaries, etc., per occupied bed upwards of £6 more than King's and upwards of £2 more than the Royal Free, the London Hospital spends upwards of £14 more than Guy's Hospital. It must be remembered, too, that Guy's Hospital in 1903 spent nearly £5 10s. per bed less upon Salaries and Wages than the average expenditure of twelve London Hospitals with medical schools in the same year. We have shown already that in Domestic Expenses Guy's has secured a reduction under a medical superintendent of £13 per occupied bed, so that the addition of this £14 saved on Salaries makes £27, or a reduction of £7 more per occupied bed under a medical superintendent than the saving claimed for this system by Sir Henry Burdett. We quote these figures for what they may be worth to illustrate that under Mr. Holland's own method of analysis the substantial accuracy of Sir Henry Burdett's contention is confirmed.*

Generally.

I entirely agree that it is necessary to bring accounts to a uniform basis before a comparison is made, and that is just what the guide has not done. I make no attack upon Guy's. I am certain that that hospital is not only well but economically managed, and nobody will regret that its diets to patients are liberal. And I know that a big hospital has to incur expenses which a small hospital is saved.

What I do object to is the insulting tone adopted by Sir Henry Burdett in the preface to the 1903 Edition of his Hospital Guide towards the London Hospital, because its committee, who may be supposed to know their own business, do not fall in with his idea of how it should be managed. He knows, he writes, that the present unfilled office of a resident superintendent is regarded with grave anxiety by many of the best friends of the London Hospital. It is curious that these friends of the "London" should flock to and complain to Sir Henry Burdett, and curious that they should be so silent to myself or to any members of our committee, 30 in number.

I am sure, too, that I only voice a very general and strong feeling against statements he has made in his letter to the Press, that any hospital which employs a medical superintendent would get down its expenditure by £20 a bed —(he said £30 at the meeting). The Guy's figures prove the contrary, and Guy's is a good example to take because by every one it is known to be a hospital carrying on its work in a thoroughly efficient manner, under a medical superintendent, too well known to need naming. I feel sure, too, that the authorities at Guy's would not support any such contention.

*All the figures in Burdett's "Hospitals and Charities" are in fact reduced to a uniform basis, so far as this can possibly be attained, with the generous co-operation of the hospital authorities, to whom all the figures are submitted before publication. The labour and expense involved are great, but they give the book its authoritative character and value.*

*We can find no reference, insulting or otherwise, to the London Hospital in the preface to the* 1903 *edition of Burdett's "Hospitals and Charities."*

*Sir Henry Burdett's statement in the Press is given above. He said at the meeting "£30 at the London. If you will let me put in a medical superintendent I will prove it." We have shown too that the facts are against Mr. Holland on this point. The figures quoted above reveal, as a matter of fact, that Guy's Hospital under a medical superintendent has cut down its expenditure by £13 per occupied bed on Domestic Expenses, and spends £14 per occupied bed on Salaries, etc., less than the London, when the figures are examined on Mr. Holland's own method, as he seems to object to the method pursued under the Uniform System of Accounts by the Editor of Burdett's "Hospitals and Charities."*

